# Update on Pathophysiology, Treatment, and Complications of Carcinoid Syndrome

**DOI:** 10.1155/2020/8341426

**Published:** 2020-01-21

**Authors:** Dominique Clement, John Ramage, Raj Srirajaskanthan

**Affiliations:** ^1^Department of Gastroenterology, Kings College Hospital London, London SE5 9RS, UK; ^2^Faculty of Life Sciences and Medicine, Kings College London, London, UK; ^3^Neuroendocrine Tumour Unit, Kings Health Partners ENETS Centre of Excellence, London SE5 9RS, UK

## Abstract

Carcinoid syndrome (CS) develops in patients with hormone-producing neuroendocrine neoplasms (NENs) when hormones reach a significant level in the systemic circulation. The classical symptoms of carcinoid syndrome are flushing, diarrhoea, abdominal pain, and wheezing. Neuroendocrine neoplasms can produce multiple hormones: 5-hydroxytryptamine (serotonin) is the most well-known one, but histamine, catecholamines, and brady/tachykinins are also released. Serotonin overproduction can lead to symptoms and also stimulates fibrosis formation which can result in development of carcinoid syndrome-associated complications such as carcinoid heart disease (CaHD) and mesenteric fibrosis. Transforming growth factor beta (TGF-*β*) is one of the main factors in developing fibrosis, but platelet-derived growth factor (PDGF), basic fibroblast growth factor (FGF2), and connective tissue growth factor (CTGF or CCN2) are also related to fibrosis development. Treatment of CS focuses on reducing serotonin levels with somatostatin analogues (SSA's). Telotristat ethyl and peptide receptor radionuclide therapy (PRRT) have recently become available for patients with symptoms despite being established on SSA's. Screening for CaHD is advised, and early intervention prolongs survival. Mesenteric fibrosis is often present and associated with poorer survival, but the role for prophylactic surgery of this is unclear. Depression, anxiety, and cognitive impairment are frequently present symptoms in patients with CS but not always part of their care plan. The role of antidepressants, mainly SSRIs, is debatable, but recent retrospective studies show evidence for safe use in patients with CS. Carcinoid crisis is a life-threatening complication of CS which can appear spontaneously but mostly described during surgery, anaesthesia, chemotherapy, PRRT, and radiological procedures and may be prevented by octreotide administration.

## 1. Introduction

Neuroendocrine neoplasms (NENs) are derived from enterochromaffin cells of the diffuse neuroendocrine system, which are mainly present in the gastrointestinal tract [1]. WHO classes all neuroendocrine cancers as neuroendocrine neoplasms (NENs) which can be divided into well-differentiated neuroendocrine tumours (NETs) and poorly differentiated neuroendocrine carcinomas (NECs) [2, 3]. Neuroendocrine neoplasm is a hormone produced in around 30–45% of cases and causes a functional syndrome. Carcinoid syndrome (CS) is the most well-known example occurring mainly in the gastrointestinal or lung-related NEN [4]. Thorsson et al. described in 1954 a series of 16 patients with a new syndrome: carcinoid syndrome, the patient symptoms of diarrhoea, skin flushing, oedema, and ascites [5].

The incidence of NENs is growing worldwide [6–8]. Based on a recent analysis of the USA Surveillance, Epidemiology, and End Results (SEER) database, this has increased from 1.03 in 1973 to 6.98 per 100.000 in 2012 [6]. Recently published United Kingdom data demonstrate a similar incidence of 8.6 per 100 000 population [9].

The prevalence of carcinoid syndrome within all NEN was reported as between 18% [10] and 72% [11] based on older series. More recent European data show a prevalence of carcinoid syndrome in 25% of 837 patients with GEP-NENs [12] and 20% of all 1263 NEN patients [13]. A recent article by Halperin et al. analyzed the SEER database in the USA, and this demonstrated that 19% of patients with neuroendocrine neoplasm had carcinoid syndrome, giving an overall incidence of carcinoid syndrome of 1 : 100 000 population [14].

In general, patients with metastatic well-differentiated NENs have a good prognosis with an overall 5-year survival rate of 50–70% depending on tumour grading, staging, and site of origin [4]. Patients with carcinoid syndrome have an overall survival of 4.7 years compared with 7.1 year in patients without symptoms of carcinoid syndrome [14].

Within this minireview, we will discuss symptoms, an update in pathophysiology, and new treatment possibilities and focus on some common complications as carcinoid heart disease, mesenteric fibrosis, and carcinoid crisis and also on more uncommon psychiatric disorders and its treatment in patients with CS.

## 2. Signs and Symptoms of Carcinoid Syndrome

Symptoms of carcinoid syndrome are diarrhoea, flushing, abdominal pain, wheezing, and palpitations, but muscle wasting is also reported [15, 16]. The most common symptom is diarrhoea which is reported in around 80% of patients [4, 17, 18]. The diarrhoea is secretory with a frequency of a least 3 bowel motions a day and is due to increased gastrointestinal motility stimulated by hormones produced by the NEN [19, 20]. Skin flushing appears in 50–85% of patients and is described as a red appearance of face, neck, and upper part of the chest which lasts from seconds to several minutes. Flushing appears as a result of hormone overproduction by NENs [4, 19–21]. Flushing may appear spontaneously but can be provoked by emotional stress, stimulation of the vagus nerve by brushing of teeth or mastication, and ingestion of alcohol or tyramine-containing foods such as cheese, coffee, chocolate, nuts, avocado, bananas, and red wine [19, 21–23]. Individuals may not be aware of the flushing episodes but often family members or friends note them [24].

Abdominal pain was described in around 40% of patients and may be related to mesenteric fibrosis [4, 15, 19]. Wheezing or bronchospasm is present in 10–20% of patients and caused by hormone overproduction [14, 19, 25–27]^.^ Histamine secretion can lead to itching and skin rashes [28]. Palpitations are a common symptom of hormone overproduction, often reported as occurring spontaneously or following a stressful episode. The palpitations can be short in duration or persist for longer periods of time, up to 30 minutes [19, 29]. Muscle wasting has been reported in small series as a result of malnutrition associated with uncontrolled diarrhoea [15]. Patients with carcinoid syndrome experience worse quality of life compared with patients with NEN without CS [30, 31]. It is important note not all diarrhoea in patients with carcinoid syndrome is hormone related.  Table 1 lists other differential diagnoses to consider patients with small bowel NENs and carcinoid syndrome.

## 3. Pathophysiology Updates

NENs may secrete as many as 40 secretory products (vasoactive substances), the most prominent being 5-hydroxytryptamine (5-HT, serotonin) but also tachykinins, histamine, kallikrein, prostaglandins catecholamines, and motilin [15, 27, 32]. It is thought these substances lead to the development of the main clinical manifestations of carcinoid syndrome (summarized in Table 2) when they reach the systemic circulation. The liver and lungs can inactivate circulating hormones, but in patients with liver metastases, the production bypasses the metabolism. Patients with ovarian, testicular or retroperitoneal metastases or those with primary bronchial tumours can rarely develop carcinoid syndrome without liver metastasis due to blood bypassing the liver when entering the systemic circulation [18, 33, 34].

**Figure 1:**
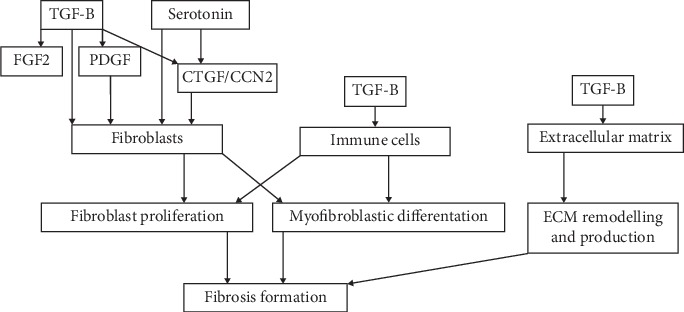
Summary of tumour microenvironment and fibrosis formation carcinoid syndrome. TGF-*β*: tumour growth factor Beta, FGF2: fibroblast growth factor, PDGF: platelet-derived growth factor, CTGF or CCN2: connective tissue growth factor.

Fibrotic complications of secreting NEN such as carcinoid heart disease and mesenteric fibrosis are the result of increased fibrosis formation within the cardiac valves and mesentery [15, 45]. One of the main factors driving fibrosis formation is thought to be serotonin since these complications occur mainly in patients with raised urine 5-HIAA, a breakdown product of serotonin [45–47].  Figure 1 summarizes the components and growth factors in the development of fibrosis formation. The tumour microenvironment (TME) consists of supportive matrix, stromal-, endothelial, and inflammatory cells, is crucial for tumour growth, invasion, and metastasis, and plays a role in the development of fibrotic complications. In Figure 1, the TME is expressed as fibroblasts, immune cells, and extracellular matrix. Fibroblasts are stimulated by serotonin but also directly and indirectly by several growth factors, transforming growth factor beta (TGF-B), platelet-derived growth factor (PDGF), basic fibroblast growth factor (FGF2), and connective tissue growth factor (CTGF or CCN2). TGF-B stimulates PDGF which has a strong proliferative effect on fibroblasts and FGF2 which has a strong mitogenic effect on fibroblasts [47]. The connective tissue growth factor is induced by serotonin and TGF-B and increases collagen synthesis, fibroblast proliferation, and differentiation into myofibroblasts [47–49]. Multiple immune cells including B- and T cells, NK cells, dendritic cells, mast cells, and tumour-associated macrophages (TAM) infiltrate the NEN tumour microenvironment creating an immunosuppressed environment, suppression of the immune response, and stimulation of fibroblast proliferation and myofibroblastic differentiation resulting in fibrosis formation. The transforming growth factor beta stimulates the third main component of TME, the extracellular matrix stimulating extracellular matrix remodelling and production which contributes to fibrosis formation [47, 49, 50].

Currently, there are no treatments to reduce fibrosis. Somatostatin analogues reduce the serotonin production by causing the tumour and have been observed in animal models of fibrotic diseases such as pulmonary- and liver fibrosis to reduce the secretion of TGF-B [51, 52]. No studies report on the role of somatostatin analogues in reducing fibrosis in carcinoid syndrome. Terguride, a serotonin receptor (5-HT2A/B) antagonist, has proven to reduce the profibrotic effects of serotonin in animals [53], in one study, in scleroderma patients, it resulted in amelioration of skin fibrosis [54]. This antifibrotic therapy has not been studied in patients with neuroendocrine neoplasms. Tamoxifen inhibits the TGF-B secretion by fibroblasts; it has shown no effect in controlling CS symptoms in case reports, but its effects on fibrotic complications have not been evaluated [55–57]. Imatinib targets the PDGF receptor and showed decreased organ fibrosis in scleroderma and pulmonary fibrosis but has not been evaluated as antifibrotic therapy [58]. Angiogenesis inhibitors such as vascular endothelial growth factor (VEGF), tyrosine kinase inhibitors, and inhibitors of the fibroblast growth factor pathways are currently been studied in patients with neuroendocrine neoplasm, but none of these studies focus on antifibrotic effects of these drugs [50]. Sulphasalazine, an anti-inflammatory drug, has showed to increase the efficacy of gemcitabine in pancreatic adenocarcinoma cells which are highly fibrotic [59]. Its anti-inflammatory effect has not been studied in neuroendocrine neoplasms or carcinoid syndrome. New research focusing on TME and growth factors may elucidate the complex process of fibrosis formation and possibly identify new targets for the treatment.

## 4. Treatment Updates

Treatment of carcinoid syndrome focuses on reducing the hormone levels or tumour load to reduced symptoms related to the syndrome. Long-acting somatostatin analogues are considered cornerstones in the treatment of carcinoid syndrome. The PROMID trial was a landmark trial demonstrating increased progression-free survival of patients with small bowel neuroendocrine tumours [60]. There was also a trend of reduced episodes of flushing in patients receiving somatostatin analogues compared with placebo. A recent meta-analysis shows symptom improvement in 65–72% of patients and biochemical response in around 45% of patients on somatostatin analogues. Increasing the dose or frequency or switching the drug type led to a reduction of symptoms in 72–84% of patients [61]. The CLARINET study randomized the GEP-NEN patients and patients with unknown primary tumour between Lanreotide autogel 120 mg and placebo and showed significantly prolonged the progression-free survival in the Lanreotide group (median not reached versus 18 months for the placebo group) [62].

Recently, telotristat ethyl, an oral inhibitor of tryptophan decarboxylase, has become available for symptomatic patients on whom treatment with somatostatin analogues is not enough to control diarrhoea. The TELESTAR trial was a phase III double-blind placebo-controlled trial in 135 patients with CS (defined as >4 bowel movements a day) who were randomized to receive 250 mg telotristat ethyl tablet, 500 mg telotristat ethyl tablet, or placebo three times daily for 12 weeks. There was a statistically significant reduction in bowel movements in 42–44% of patients (250 mg or 500 mg) compared with 20% reduction in placebo patients after 12 weeks. A significant reduction in the urine-5 HIAA level and improvement in quality of life scores was seen [63, 64]. A companion trial (TELECAST) looking at 126 patients with CS and less than 4 bowel motions a day and with either loose stools or daily >2 flushing episodes or abdominal pain or nausea in >20% of days or 5-HIAA urine levels above normal limits classified 40% of patients on telotristat ethyl as durable responders [65]. Another study published by Weickert et al. investigated the nutritional status of 120 patients participating in the TELESTAR study; weight gain and improvement in nutritional markers (albumin, cholesterol levels and triglycerides) were seen after 12 weeks in patients on Telotristat ethyl but not in patients on placebo [66].

In patients with progressive disease on somatostatin analogues, peptide receptor radionuclide therapy can be considered. Somatostatin receptors are highly expressed in well-differentiated neuroendocrine neoplasm which is a target for peptide receptor radionuclide therapy (PRRT), a radiolabeled somatostatin analogue therapy. Lutetium-177-DOTATATE (^177^Lu) is a beta- and gamma-emitting radionuclide and was studied in the NETTER 1 study, wherein 221 patients with metastasized or locally advanced midgut NENs were randomized between ^177^-Lu-DOTATATE- or octreotide LAR 60 mg every 4 weeks. The interim analysis after 20 months showed progression-free survival for 65.2% of patients treated with ^177^-Lu-DOTATATE and 10.8% of patients treated with octreotide LAR [67]. The health-related quality of life from patients treated with PRRT in the NETTER-1 study was recently published, and this demonstrated an improvement in symptoms and delay in deterioration of quality of life scores in patients receiving PRRT compared with high-dose somatostatin analogue alone [68]. Furthermore, another study has examined the quality of life and was studied in 50 patients with metastatic neuroendocrine neoplasms before and 6 weeks after finishing 4 cycles of ^177^-Lu-DOTATATE, and it showed significant improvement of functional and quality of life scores [69]. Limited data exist concerning the role of PRRT in patients with refractory carcinoid syndrome without evidence of radiologically progressive disease. The aforementioned meta-analysis shows 64–74% of symptom improvement based on 4 small-phase II studies [61]. Two small studies with 11 and 14 NEN patients with carcinoid syndrome report a good effect of ondansetron in reducing diarrhoea [70, 71].

**Figure 2:**
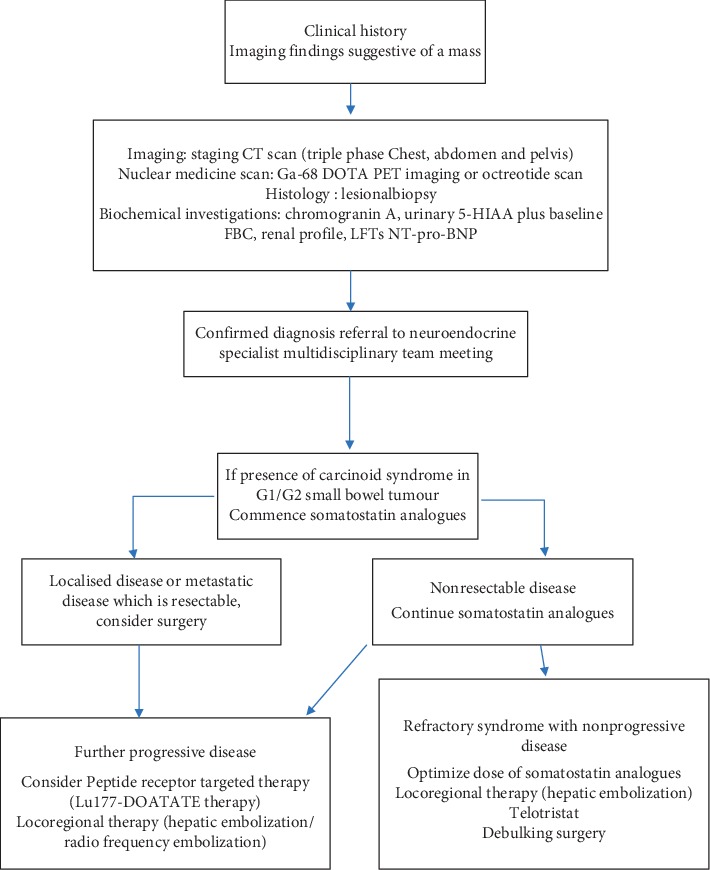
Proposed algorithm for management of carcinoid syndrome. NEN: neuroendocrine tumour; LFTs: liver function tests; FBC: full blood count.

There are a number of guidelines published to aid in the determining the stepwise management approach for patients with NENs.  Figure 2 illustrates a proposed treatment algorithm for patients with carcinoid syndrome, with a focus on first-line therapy and then options available for second-line treatment.

### 4.1. Complications: Carcinoid Heart Disease

The prevalence of carcinoid heart disease is estimated in modern series to be around 20% of patients with carcinoid syndrome, but this is higher in older series, with rates up to 60% [72, 73]. Carcinoid heart disease develops as a result of fibrous plaques (as explained in Figure 2) depositing on cardiac valves and endocardial thickening usually on the right side of the heart. This plaque formation leads to retraction and fixation of the leaflets of the tricuspid and pulmonary valves. The main findings are tricuspid regurgitation or stenosis and pulmonary stenosis, and both valves can be involved in 31% of patients. Left-side heart disease can occur in less than 10% of patients mainly because of a patent foramen ovale [15, 18, 74, 75].

Carcinoid heart disease may be asymptomatic but may present as fatigue and progressive exertional dyspnoea. Progressive development of heart failure can occur and eventually severe heart failure presenting with oedema, weight gain, ascites, and right upper abdominal pain [15, 18, 74, 76].

The diagnosis is made by echocardiogram to evaluate valve leaflets, valves, atrial or ventricular dilatation, and endocardial thickening [15, 76, 77]. The ENETS guidelines advise the annual echocardiographic screening in patients with carcinoid syndrome and signs of carcinoid heart disease [78]. The UKINETS guidelines advise screening with serum N-terminal pro-brain natriuretic peptide (NT-proBNP), a surrogate marker of carcinoid heart disease [79]. Patients should be referred for echocardiography with a serum NT-proBNP concentration >260 picogram/ml [76, 79–81]. Carcinoid heart disease is associated with limited 3-year survival of 31% compared with patients with metastatic neuroendocrine tumour without carcinoid heart disease of 68% [76, 82, 83]. Risk factors for development or progression of carcinoid heart disease are 5-HIAA levels ≥300 micromol/24hous, ≥ 3 flushing episodes per day, or treatment with cytotoxic chemotherapy [72, 84].

Valve replacement surgery or valvuloplasty is the only definitive treatment and should be planned carefully and early in the course of the disease [76, 85]. The choice of valve prosthesis is still under debate: bioprosthesis is preferred to mechanical valves, due to the absence of need for anticoagulation and the risk of bleeding complications, but these valves may be less durable and more vulnerable for the recurrence of disease. Postoperative management should focus on controlling hormone levels to prevent recurrent disease [77]. The perioperative mortality since 2000 is around 5% [86, 87]. After surgery, the one-year survival is 70% and at 5 years, it was 40% [86, 87]. It is hypothesized that reducing serotonin levels may prevent the development of carcinoid heart disease and the need for valve replacement surgery and new treatments such as telotristat ethyl and PRRT may be contributing to this [15, 72, 78].

### 4.2. Complications: Mesenteric Fibrosis

Small bowel neuroendocrine tumours frequently metastasize to lymph nodes in the mesentery. The NEN cells within these lymph nodes can produce hormones and growth factors which can result in mesenteric fibrosis [48, 88]. The exact mechanism of fibrosis has not been elucidated but the role of several factors is explained in Figure 2. The fibrosis causes shrinkage and fixation of the mesentery and mesenteric root to the retroperitoneum leading to small bowel obstruction. The mesenteric vessels may also become entrapped or occluded with resulting venous stasis and ischemia and often impairment of the arterial and venous circulation. The fibrosis can extend to the retroperitoneum and cause stenosis of the ureters and hydronephrosis.

Interestingly, a recent study by Blažević et al. [89] suggested that possibly female sex hormones may have a role in reducing mesenteric mass formation. This was based on the finding that mesenteric mass lesions were less commonly seen in women ≤52 years of age than compared with men. In view of the risk of ischemia or small bowel obstruction, removal of the primary tumour and mesenteric mass is advised when possible [15, 78, 88, 90]. Two recent studies [89, 91] investigated the effect of mesenteric fibrosis diagnosed on CT scans and survival. Blažević et al. [89] showed that mesenteric fibrosis was present in up to 41.4% of patients with biopsy-proven small bowel NEN visible on CT scan. Mesenteric fibrosis was not a prognostic factor of overall survival with a median survival of 8.7-year and 5-year survival of 71%. They showed no survival benefit for palliative surgery, removal of mesenteric mass, or prophylactic surgery although Laskaratos et al. [91] showed primary resection was associated with a longer overall survival. In both studies, older age, higher 24 h urine 5-HIAA levels, and chromogranin A levels were associated with the development of mesenteric fibrosis.

### 4.3. Complications: Psychiatric Disorders

Serotonin is a neurotransmitter and involved in multiple processes within the central nervous system (CNS) such as mood, behavior, and sleep, but it cannot pass the blood-brain barrier. The CNS serotonin production is dependent on serotonin precursor tryptophan. In CS, there is a shift towards tumour-derived serotonin production resulting in CNS tryptophan deficiency. This relative tryptophan deficiency can contribute to the development of cognitive problems [92]. In an older series from the 1970s, screening medical records for symptoms referring to psychiatric symptoms, up to 50% of 20 patients with CS were suffering from depression, 35% of anxiety disorders, and 35% of confusion [93]. A survey of NEN patients in the USA (27% of total 186 patients with carcinoid tumours) showed 3.8% of patients fulfilled the criteria for depression and 18.3% of patients are at risk for depression based on the hospital anxiety and depression score (HADS) [94]. More recent studies screening patients with CS and without CS using HAD scores report 12% of patients with depression [30]. Other hormonally active substances released by the NEN which can pass the blood-brain barrier could also contribute to cognitive problems [95].

Two studies describe cognitive impairment in patients with carcinoid syndrome. Russo et al. screened 14 CS patients and 14 healthy volunteers with neuropsychological assessment and showed rapid visual information processing was impaired compared with healthy volunteers while the ability to attend to the specific attributes of compound stimuli and to shift that attention when required was better [92]. Chambers et al. evaluated cognitive impairment in 21 patients with CS with self-reported questionnaires (MASQ symptom scores) and neurocognitive assessment and compared the outcomes with norm scores for healthy volunteers. Patients with CS reported higher MASQ scores for verbal memory, attention and concentration, language, visual memory, and executive function. In the neurocognitive assessment, nearly all scores for these tasks were all within normal range except from verbal memory and visual perception function [95].

One of the most used antidepressants is selective serotonin reuptake inhibitors (SSRIs). Several case reports described increase of CS and other symptoms in patients with carcinoid, related to the use of citalopram, fluoxetine, or sertraline. The authors caution against the use of SSRIs in patients with CS [96–99]. Two recent retrospective studies describe patients with carcinoid syndrome and the use of antidepressants. Shi et al. report on 52 patients (48 patients with carcinoid syndrome and 4 without), and a total of 73 antidepressants trailed. In 6 patients (8.7%), there was an increase in CS symptoms within 3 months after starting but no reason to discontinue the antidepressants. None of the patients developed a carcinoid crisis or other medical emergency [100]. Isenberg-Grzeda et al. describe 92 patients with metastatic NEN (76 without CS and 16 with CS) and the use of antidepressants. In their cohort, the mean time on antidepressant was 1 year and 50% of patients stopped due to remission of symptoms. No cases of carcinoid crisis were reported, and there is no correlation with clinical symptoms or neuroendocrine neoplasm markers such as 5-HIAA in urine [101].

Inhibition of tryptophan hydroxylase to reduce symptoms of carcinoid syndrome had depression as a major side effect [102]. A recently developed oral small-molecule TPH inhibitor (Telotristat ethyl) which cannot pass the blood-brain barrier was studied in 2 randomized phase III placebo-controlled trails (TELESTAR and TELECAST) in patients with carcinoid syndrome with special interest for depression as side effect [63, 66]. Within the TELESTAR, trial depressive symptoms were recorded in 6.7% of patients within the placebo and 250 mg groups versus 15.6% in the 500 mg group. None of the patients needed new antidepressant therapy, and none of the cases resulted in discontinuation of therapy [63]. In the TELECAST trial, depressed mood was present with 7.7% of patients in the placebo group, 4.0% of patient in the 250 mg group, and none in the 500 mg group. During the open label extension period, 3.0% of all patients reported a depressed mood. Only 2 patients stopped with telotristat ethyl due to depressive symptoms, but both had an underlying depression and were using antidepressants at baseline [65].

Depression and anxiety are common psychiatric complication among patients with CS and deserve attention from clinicians. Prospective studies could provide more evidence regarding the use of SSRIs. Two small retrospective studies reported that SSRIs did not provoke exacerbation of symptoms or carcinoid crisis. Longitudinal studies with higher numbers of CS patients could provide more information about cognitive impairment. The two current studies point in this direction, but details are limited.

### 4.4. Complications: Carcinoid Crisis

A carcinoid crisis is a serious and potentially life-threatening exacerbation of the carcinoid syndrome, characterized by profound flushing, bronchospasm, tachycardia, and widely fluctuating blood pressure. There is no consensus about the precise definition: retrospective and prospective studies on this topic used different definitions [15, 103–106]. The main symptoms are severe flushing, bronchospasm, profound hypotension and arrhythmias or hypertension, central nervous system disfunction (stupor and confusion), and diarrhoea. The current hypothesis is that a rapid release of vasoactive hormones by NEN cells will cause a crisis. A recent study on 46 patients with carcinoid syndrome having abdominal surgery could not confirm this hypothesis: no massive release or changes in serotonin, histamine, kallikrein, or bradykinin were observed in patients having a hypotensive episode during surgery [107]. Carcinoid crisis appears spontaneously but can be precipitated by surgery, anaesthesia, chemotherapy, PRRT, radiological procedures, and stress. Two recent studies show differences in the prevalence of carcinoid crisis in a retrospective study, 3.4% of 150 patients with small bowel NENs on octreotide cover [105] compared with 30% in a prospective series of 127 patients with carcinoid tumour of which 74% had carcinoid syndrome [103]. Lack of appropriate treatment can result in death.

Octreotide cover has been advised to prevent the development of carcinoid crisis, but the ideal schedule has not been established [108]. More recently, several studies have tried to find a regime preventing carcinoid crisis. In the first published retrospective study on this topic, none of patients with carcinoid tumours who received pre- and/or intraoperative octreotide (dose 300–350 *μ*g) cover developed intraoperative complications versus 18% of patients who did not receive octreotide cover [106]. A single preoperative dose of 500 *μ*g octreotide did not prevent 24% of patients with carcinoid tumour developing carcinoid crisis [109]. A preoperative bolus with 500 *μ*g octreotide bolus followed by an intraoperative infusion at a rate of 500 *μ*g octreotide per hour prevented 70% of patients with carcinoid tumours from developing carcinoid crisis [103]. Octreotide cover with pre-, intra-, and postoperative octreotide 500 *μ*g/hour intravenously led to a carcinoid crisis in only 3.4% of patients with small bowel NENs having cytoreductive surgery [105].

The ideal prophylactic octreotide scheme and dose is not clearly established, several guidelines advise on different schedules. The ENETS guideline advises a schedule with octreotide 50–100 *μ*g/h intravenously 12 before up to 48 h after surgery [104]. The North American neuroendocrine tumour society (NANETS) guideline advises that routine administration of octreotide does not prevent a carcinoid crisis but advises a schedule of octreotide 100–500 *μ*g/h intraoperatively [110]. In contrast, the UKINETS guideline [111] provides an extensive schedule on different patient categories and type of procedures for dose and period of octreotide cover.

### 4.5. Complications: Skin and Pulmonary Fibrosis and Pellagra

Several small series describe skin scleroderma associated with carcinoid syndrome. Serotonin and other hormones causing recurrent flushing and vasospasm resulting in a local inflammatory response are believed to be the causative agents [15]. Pulmonary fibrosis in patients with carcinoid syndrome is described in a small series of 14 patients. The pathophysiologic mechanism in unclear since 5-HIAA levels of patients with pulmonary fibrosis did not differ from levels of patients without pulmonary fibrosis [112].

Pellagra as a result of tryptophan deficiency with symptoms of dermatitis, diarrhoea, and dementia can develop in about 5% of patients with carcinoid syndrome [17, 23, 113]. The dermatitis is bilateral and symmetrical on areas exposed to sunlight, heat, friction, or pressure. It begins as an erythema with acute or intermittent onset gradually changing to an exudative eruption on the dorsa of the hands, face, neck, and chest with pruritus and burning. Initial bright red well‐demarcated erythema changes to cinnamon‐brown in color [114]. Niacin levels in blood and urine are difficult to measure [115, 116]. Nicotinamide 100–200 mg three times a day should be prescribed if pellagra is suspected.

## 5. Conclusion

Carcinoid syndrome can develop in patients with hormone producing neuroendocrine neoplasms. Several hormones are involved in the development of symptoms and (fibrotic) complications. New treatments such as telotristat and PRRT to reduce hormone levels have become available and whether they are able to prevent fibrotic complications is a topic for future research. Antifibrotic treatments are also a major topic for further research. Current data report conflicting outcomes for the role of prophylactic surgery in patients with mesenteric fibrosis. Depression, anxiety, and cognitive impairment are reported symptoms in patients with carcinoid syndrome, and two recent studies show safe use of SSRI's in these patients. Carcinoid crisis is a life-threatening complication of carcinoid syndrome, and there are several definitions to describe this crisis. Prophylaxis with intravenous or subcutaneous octreotide has been described in different doses and schedules; further research could find an optimal regime. Despite being recognised for over 65 years, carcinoid syndrome is still not well understood and future studies are needed to understand the various devastating effects it can have on body systems.

## Figures and Tables

**Table 1:** The differential cause of worsening diarrhoea in patients with carcinoid syndrome.

Cause	Investigation to confirm	Treatment
Post-bowel surgery (i) Bile salt malabsorption (ii) Short bowel syndrome	SeCHAT scan CT enterography, biochemical factors: low magnesium and low urinary sodium	Bile acid binding resins PPI, loperamide, codeine IV fluids

Post-pancreatic resection (i) Pancreatic exocrine Insufficiency (ii) Bacterial overgrowth	Faecal elastase Breath tests Breath tests for glucose and lactulose	Pancreatic exocrine replacement therapy Antibiotics

Irritable bowel syndrome	Exclusion of other causes	FODMAP diet Probiotics

Other diagnoses (i) Inflammatory bowel disease (ii) Microscopic colitis		

**Table 2:** Vasoactive substances, mechanism of action, and clinical symptoms in carcinoid syndrome.

Name	Mechanism of action	Associated with
Serotonin (5-HT) [21, 35]	Vasoconstriction or vasodilatation Increased gut motility Increased secretion of water, sodium, chloride, and potassium	Diarrhoea, flushing, palpitations

Tachykinins (substance K, substance P, neuropeptide K, neurokinin A) [36–38]	Cutaneous vasodilatation, tachycardia, hypotension, and increased small intestine motility	Diarrhoea, flushing, palpitations

Histamine [5, 39]	Vasodilatation	Flushing, wheezing, palpitations

Kallikrein [40]	Release/stimulation of bradykinin resulting in hypotension	Flushing, palpitations

Prostaglandin [41]	Increased intestinal motility and fluid secretion Vasodilatation	Diarrhoea, flushing, palpitations

Catecholamines (norepinephrine) [42, 43]	Initiate cascade resulting in serotonin release	Flushing, palpitations

Motilin [44]	Initiation of interdigestive migrating motor complex (IMMC)	Diarrhoea

## Abbreviations

5-HT: 5-Hydroxytryptamine or serotonin
CaHD: Carcinoid heart disease
CS: Carcinoid syndrome
CTGF or CCN2: Connective tissue growth factor
ENETS: European Neuroendocrine Tumour Society
FGF2: Fibroblast growth factor
IV: Intravenous
MASQ: Multiability self-questionnaire
NANETS: North American Neuroendocrine Tumour Society
NEC: Neuroendocrine carcinoma
NEN: Neuroendocrine neoplasm
NET: Neuroendocrine tumour
NT-proBNP: N-terminal pro-brain natriuretic peptide
PDGF: Platelet-derived growth factor
PRRT: Peptide receptor radionuclide therapy
SEER database: Surveillance, Epidemiology, and End Results database
SSA's: Somatostatin analogues
SC: Subcutaneous
SSRI: Selective serotonin reuptake inhibitor
TGF-*β*: Tumour growth factor beta
TME: Tumour microenvironment
UKINETS: United Kingdom Neuroendocrine Tumour Society.
